# Neurocardiac Crosstalk: Sympathetic Remodeling and Arrhythmogenesis After Myocardial Infarction

**DOI:** 10.3390/cimb47121037

**Published:** 2025-12-12

**Authors:** Tianshui Yu

**Affiliations:** Key Laboratory of Evidence Science, China University of Political Science and Law, Ministry of Education, Beijing 100088, China; cu007956@cupl.edu.cn

**Keywords:** myocardial infarction, sympathetic remodeling, ventricular arrhythmias, paraventricular nucleus, rostral ventrolateral medulla, nucleus of the solitary tract, TLR4/MyD88/NF-κB pathway, P2X7R/NLRP3 pathway, GABA/GABA receptors pathway

## Abstract

Sympathetic remodeling following myocardial infarction (MI) is a critical mechanism underlying the development of malignant arrhythmias and sudden cardiac death (SCD). The cardiac sympathetic nervous system functions as a multi-level regulatory network, integrating centers from the cerebral cortex (e.g., the insular lobe and anterior cingulate gyrus), subcortical structures (e.g., the paraventricular nucleus of the hypothalamus), and brainstem nuclei (e.g., the rostral ventrolateral medulla and nucleus of the solitary tract), down to the peripheral ganglia. Post-MI, this entire neural axis undergoes significant remodeling, which manifests as neuroinflammation in the central nervous system, alongside peripheral sympathetic nerve sprouting and heterogeneous hyperinnervation. This article provides a systematic review of the anatomical architecture of the cardiac sympathetic nerve and the regulatory mechanisms of sympathetic remodeling at various levels of the central nervous system after MI. It particularly focuses on key signaling pathways—including the TLR4/MyD88/NF-κB and P2X7R/NLRP3 inflammasome pathways, as well as GABAergic inhibition within the paraventricular nucleus—in addition to the peripheral remodeling mechanisms within the stellate ganglia. By synthesizing insights from these studies, this review offers a novel perspective for understanding the neuroimmune mechanisms of post-MI malignant arrhythmias and provides a theoretical foundation for elucidating the mechanisms of SCD in clinical practice.

## 1. Introduction

Myocardial infarction (MI) remains a leading cause of global mortality. According to the latest Global Burden of Disease Study, cardiovascular diseases were responsible for approximately 19.8 million deaths worldwide in 2022, with ischemic heart disease constituting the majority of these cases [1]. The global prevalence of MI is estimated at 3.8% in individuals under 60 years and rises to 9.5% in those over 60 [2]. MI is triggered by an acute occlusion of a coronary artery, leading to an interruption of blood and oxygen supply to the downstream myocardium. This results in cardiomyocyte necrosis, followed by an inflammatory response, fibrosis, and eventual scar formation [3]. These pathological changes not only impair cardiac function, often culminating in heart failure, but also create an electrophysiological substrate of heterogeneity that serves as a critical matrix for malignant ventricular arrhythmias (VAs) and sudden cardiac death (SCD) [4,5].

The cardiac autonomic nervous system, particularly the sympathetic branch, plays a pivotal role in the post-MI pathophysiology. Under physiological conditions, a balance between sympathetic and parasympathetic tones maintains dynamic cardiac control [6]. Following MI, however, the sympathetic nervous system undergoes significant remodeling. This process is characterized by denervation in the infarct and peri-infarct zones, aberrant sympathetic nerve sprouting, and an increased heterogeneity in nerve distribution density [7,8,9,10]. This sympathetic remodeling follows a time-dependent trajectory: acute phase arrhythmias (hours to days post-MI) arise from ischemia-induced electrophysiological instability, while subacute to chronic arrhythmias (weeks to months) result from structural remodeling including sympathetic nerve sprouting, which typically peaks within three months [11]. The mechanisms reviewed here primarily address the subacute-to-chronic sympathetic remodeling contributing to sustained arrhythmia risk and heart failure progression. Most of the regenerated nerve fibers are tyrosine hydroxylase—positive, indicating a predominance of mature sympathetic nerves [12]. The resulting spatial and transmural heterogeneity disrupts action potential durations and prolongs the QT interval, thereby establishing an electrophysiological foundation for re-entrant arrhythmias [13,14].

In recent years, the brain–heart axis perspective has emerged as a novel paradigm for investigating the neural mechanisms underlying cardiovascular diseases. The brain–heart axis constitutes a bidirectional communication network between the heart and the brain, involving integrated neural, hormonal, and immune pathways [15,16,17]. After MI, ischemic injury signals are relayed via afferent nerves to various levels of the central nervous system (CNS), triggering a neuroimmune response. The consequent CNS overactivation, in turn, promotes cardiac sympathetic remodeling through efferent pathways, creating a vicious cycle. This comprehensive reorganization of the cardio-brain axis is now recognized as a crucial mechanism in the pathogenesis of post-infarction VAs [7,18,19].

Current research on post-MI sympathetic remodeling has primarily focused on local cardiac mechanisms. In contrast, the regulatory roles of upstream centers—specifically, the interactions among different levels of the CNS and the mechanisms by which higher brain centers influence sympathetic remodeling—are not fully elucidated. This article systematically reviews the anatomy of the cardiac sympathetic nervous system and the regulatory mechanisms of sympathetic nerve regeneration and remodeling by central centers, from the higher cortex to the brainstem and peripheral ganglia, following MI. We particularly highlight key signaling pathways within the paraventricular nucleus (PVN) and their association with VAs, and discuss the implications of these mechanisms for determining the cause of SCD in medical practice.

## 2. Methods

This narrative review synthesizes current literature on sympathetic remodeling and arrhythmogenesis following myocardial infarction, with particular emphasis on the brain–heart axis. A comprehensive literature search was conducted using PubMed, Web of Science, Google Scholar, and Embase. Search terms included combinations of “myocardial infarction,” “sympathetic remodeling,” “ventricular arrhythmias,” “paraventricular nucleus,” “rostral ventrolateral medulla,” “nucleus of the solitary tract,” and key signaling pathways (TLR4/MyD88/NF-κB, P2X7R/NLRP3, GABA). We prioritized original research articles and reviews that provide mechanistic insights into the central and peripheral regulation of cardiac sympathetic activity post-MI.

## 3. The Multi-Level Anatomical Structure of the Cardiac Sympathetic Nervous System

### 3.1. Cardiac Nervous System

The heart is innervated by a sophisticated and highly organized neuroanatomical network. The cardiac autonomic nervous system plays an essential role in regulating cardiac function—including heart rate, contractility, and conduction velocity—and its dysregulation is implicated in various cardiovascular pathologies, such as arrhythmias [20,21]. Anatomically, the cardiac autonomic system is divided into extrinsic (extracardiac) and intrinsic (intracardiac) components. The extrinsic system mediates communication between the heart and the CNS as well as other peripheral nerves, while the intrinsic system consists of autonomic nerve fibers and ganglionated plexuses located within the pericardium [22].

#### 3.1.1. Extracardiac Nervous System

The extracardiac nervous system comprises structurally and functionally distinct sympathetic and parasympathetic nerve fibers. While these two divisions are largely separate, limited interconnections exist between them. This system originates from the CNS—notably the insular cortex—descends through the medulla, and ultimately connects with the intracardiac nervous system. Cardiac sympathetic innervation arises from the superior, middle, and inferior cervical ganglia of the bilateral sympathetic trunks, which align with segments from the cervical to the upper thoracic spinal cord. The superior cervical ganglia correlate with spinal levels C1–C3, while the inferior cervical ganglion is associated with C7–C8 to T1–T2. The cell bodies of postganglionic neurons are located primarily within the stellate ganglion and the cervical ganglia. The stellate ganglion, situated at the transverse process of the C7 vertebra and anterior to the first rib, plays a pivotal role in cardiac sympathetic innervation. Its postganglionic fibers contribute to the superior, middle, and inferior cardiac nerves, which collectively form an intricate neural network known as the cardiac plexus. These sympathetic nerves distribute to key cardiac structures including the sinoatrial node, atrioventricular node, and the main trunks of the left and right coronary arteries. They follow the coronary arteries into the ventricular myocardium, course along the long axis of cardiomyocytes, and ultimately terminate in the endocardium. Upon activation, sympathetic fibers release norepinephrine, which binds primarily to β_1_-adrenergic receptors on cardiomyocytes. This interaction enhances calcium influx during action potentials, thereby increasing cardiac contractility (positive inotropy) [23,24,25,26,27].

The cardiac parasympathetic nerves originate from multiple sources: the cervical cardiac branches of the bilateral vagus nerves, the cervicothoracic cardiac branches (which may include contributions from the right recurrent laryngeal nerve), and the thoracic cardiac branches of the vagus along with the left recurrent laryngeal nerve. These nerves are divided into superior, middle, and inferior branches, which intertwine with sympathetic fibers at the inferior border of the aortic arch and anterior to the tracheal bifurcation, forming the superficial and deep cardiac plexuses. From these plexuses, branches extend into the heart [28]. The postganglionic parasympathetic fibers predominantly innervate the sinoatrial node, atrioventricular node, atrial myocardium, and coronary arteries [29]. When activated, these fibers release neurotransmitters including acetylcholine and vasoactive intestinal peptide. ACh binds to M_2_ muscarinic receptors, inducing negative chronotropic and inotropic effects [30].

The functional significance of parasympathetic and sympathetic innervation in cardiovascular regulation and neuroimmune communication has been established through decades of seminal investigations. Early neurophysiological studies demonstrated that myocardial ischemia excites afferent cardiac sympathetic nerve fibers, which relay injury signals to central autonomic centers [31]. Subsequent anatomical studies revealed differential vulnerability of autonomic pathways: phenol applied to the ventricular epicardium selectively interrupts sympathetic afferents while sparing vagal pathways, indicating distinct anatomical trajectories of these two systems [32]. This differential sensitivity has important implications for understanding how MI-induced damage affects autonomic signaling.

The role of vagal pathways in immune-to-brain communication has been firmly established. Systemic administration of inflammatory cytokines such as IL-1β induces synthesis of pro-inflammatory mediators in hypothalamic nuclei, an effect completely blocked by subdiaphragmatic vagotomy, demonstrating that vagal afferents serve as a critical conduit for peripheral immune signals to reach central autonomic centers [33]. The blood–brain barrier, while restrictive, does permit passage of certain cytokines through saturable transport mechanisms, providing an additional route for neuroimmune communication [34]. Following acute MI, injury signals rapidly trigger hypothalamic cytokine synthesis, including TNF-α and IL-1β, indicating that cardiac damage activates central neuroimmune responses within hours [35,36]. Importantly, central mineralocorticoid receptor blockade attenuates this post-MI increase in plasma TNF-α, revealing that central neuroendocrine pathways actively regulate peripheral inflammatory responses after cardiac injury [37].

The intrinsic cardiac nervous system, comprising extensive ganglionated plexi, represents a site where neuroimmune interactions directly modulate cardiac function. Administration of neuropeptides to intrinsic cardiac neurons elicits complex cardiac responses, demonstrating that these local neural circuits integrate not only classical neurotransmitter signals but also peptidergic modulation [38]. In the context of heart failure, the interaction between baroreceptor inputs, vagal afferents, and sympathetic efferents becomes dysregulated. Studies in chronic heart failure models demonstrate that both baroreceptor activation and vagal afferent stimulation can inhibit cardiac sympathetic afferent reflexes and reduce sympathetic nerve activity, highlighting potential therapeutic targets for restoring autonomic balance [39].

Critically, post-MI parasympathetic withdrawal—reflected clinically in reduced heart rate variability and impaired baroreflex sensitivity—removes these protective inhibitory influences. The inflammatory milieu post-MI affects not only the myocardium but also the autonomic nerves themselves, leading to altered neurotransmitter synthesis and remodeling of both sympathetic and parasympathetic innervation patterns. Thus, the sympathetic-parasympathetic imbalance, rather than sympathetic hyperactivity alone, constitutes a critical mechanism underlying post-MI arrhythmogenesis. Understanding these bidirectional neuroimmune interactions provides a foundation for developing therapeutic strategies that target both central and peripheral components of the autonomic nervous system.

#### 3.1.2. Intracardiac Nervous System

Accumulating evidence indicates the presence of a sophisticated autonomic network within the heart itself, often termed the heart’s “little brain”. This intrinsic cardiac nervous system comprises numerous ganglia that form synaptic connections with extrinsic sympathetic and parasympathetic nerves entering the pericardium. Each ganglion contains between 200 and 1000 neurons [40]. Anatomically, in the adult human heart, all intracardiac ganglia are located within the atrial regions. Larger ganglia are predominantly clustered around the sinoatrial and atrioventricular nodes, while smaller ganglia are distributed across the superior surface of the left atrium, the interatrial septum, and the junctions between the atrial appendages and the atrial body [41]. The vast majority of these ganglia are interconnected via extensive networks of interneurons, forming ganglionated plexi (GP) primarily embedded within the epicardial fat pads and cardiac tissue [42,43]. These GPs serve as critical integration centers, where extrinsic inputs from the extracardiac nervous system converge. This organization allows the intracardiac nervous system to process information and exert local control, thereby fine-tuning the complex interplay between intrinsic and extrinsic autonomic influences on cardiac function [44,45].

#### 3.1.3. Regulation of Cardiac Function by the Cardiac Nervous System

**Figure 1:**
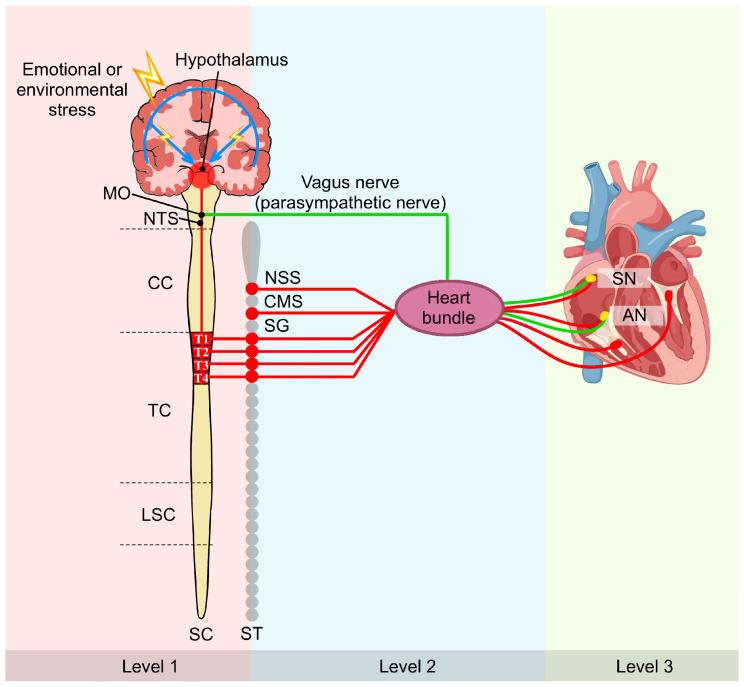
Multi-tiered regulation of cardiac function by the cardiac nervous system: a brain–heart axis perspective. This schematic illustrates the three-level hierarchical organization of cardiac autonomic control. Level 1 comprises central neurons within the MO and SC, which are modulated by higher cortical structures. Level 2 consists of peripheral intrathoracic extracardiac ganglia, including the SG. Level 3 represents the intrinsic cardiac nervous system, which includes GP on the heart itself. Sympathetic nerves (red) and parasympathetic nerve (green) converge and integrate within the cardiac muscle and GP. This hierarchical framework establishes the anatomical foundation for understanding how MI-induced remodeling propagates across multiple neural levels, from cortical neuroinflammation to peripheral nerve sprouting, ultimately creating the substrate for post-MI arrhythmias. Symbol explanations: Lightning bolt (⚡): Emotional or environmental stress. Abbreviations: AN, atrioventricular node; CC, cervical cord; CMS, cardiac motor system; GP, ganglionated plexi; LSC, lumbar spinal cord; MO, medulla oblongata; NSS, nucleus of the solitary system; NTS, nucleus of the solitary tract; SC, spinal cord; SG, stellate ganglion; SN, sinoatrial node; ST, sympathetic trunk; TC, thoracic cord. Figure is original artwork created for this review.

The cardiac autonomic system employs hierarchical control across three levels [46]: central neurons (medulla and spinal cord, modulated by cortical centers), intrathoracic extracardiac ganglia (stellate and cervical ganglia), and the intrinsic cardiac nervous system. The ICNS possesses local autonomy through bipolar neurons forming reflex circuits that operate independently of central input [47,48]. Dysregulation of this system contributes to cardiac arrhythmias (Figure 1).

### 3.2. Cardiac Sympathetic Nerve Innervation System

The heart is regulated by a sophisticated, multi-tiered sympathetic nerve network that integrates central and peripheral components. Functionally, this system can be divided into four principal levels: (1) cortical integration centers, including the insular cortex, anterior cingulate cortex, and medial prefrontal cortex, process emotional and cognitive signals to modulate cardiovascular activity; (2) higher subcortical autonomic centers, notably the PVN of the hypothalamus, serve as key hubs for autonomic integration, receiving cortical inputs and projecting to brainstem autonomic regions; (3) primary cardiovascular centers in the brainstem, such as the rostral ventrolateral medulla (RVLM) and the nucleus tractus solitarius (NTS), which directly regulate sympathetic outflow to the heart; (4) peripheral ganglia, primarily the stellate ganglia, where preganglionic fibers synapse with postganglionic neurons that innervate the heart.

Among these, the PVN acts as a critical subcortical center, integrating inputs from cortical areas and modulating cardiac sympathetic activity via projections to the RVLM and NTS.

#### 3.2.1. Paraventricular Nucleus (PVN)

The hypothalamus serves as a central regulator of neuroendocrine and autonomic functions. Research indicates that it plays a pivotal role in modulating cardiovascular processes by releasing neurohormones—such as thyrotropin releasing hormone, corticotropin releasing hormone, oxytocin, vasopressin, and somatostatin—and integrating homeostatic feedback mechanisms via autonomic signaling pathways [49,50]. Within the hypothalamus, the PVN, situated in the medial anterior hypothalamus along the lateral wall of the third ventricle, stands out as one of the most critical nuclei. Neurons in the parvocellular division of the PVN project to autonomic centers in the brainstem and spinal cord, thereby influencing sympathetic pathways involved in cardiovascular regulation. Furthermore, parvocellular neurons release a variety of neurotransmitters, including excitatory mediators like glutamate and NE, as well as inhibitory signals such as nitric oxide and gamma-aminobutyric acid (GABA), which collectively fine-tune cardiovascular activity. This neurotransmitter balance is essential for maintaining normal cardiac function. Studies have shown that a reduction in inhibitory neurotransmission (e.g., GABA) within the PVN can lead to relative dominance of excitatory signals like glutamate, resulting in sympathetic overactivation [47]—a finding consistent with the pathophysiological mechanisms underlying certain cardiovascular disorders.

#### 3.2.2. Rostral Ventrolateral Medulla (RVLM)

The medulla oblongata serves as a pivotal cardiovascular control center in the human brain, functioning as both a relay for descending autonomic pathways and a site for reflex circuits that regulate blood pressure and cardiac performance. Within the medulla, the RVLM plays a particularly critical role. It contains premotor glutamatergic neurons that excite sympathetic output by integrating inputs from higher brain regions to set sympathetic tone. These neurons receive afferent projections from the hypothalamic PVN and send efferent fibers caudally to the intermediolateral cell column (IML) of the thoracic spinal cord (T1–T5). Here, preganglionic sympathetic neurons synapse with noradrenergic neurons in the cervical and stellate ganglia, which in turn innervate the heart and adrenal medulla to directly modulate cardiovascular function and catecholamine release. Following MI, the heart undergoes significant neural remodeling, where alterations in extracellular matrix composition influence sympathetic reinnervation patterns [51]. Experimental studies have demonstrated that MI in rats enhances sympathetic nerve activity from PVN neurons projecting to the RVLM, highlighting its role in post-injury autonomic dysregulation [52]. Additionally, a subset of RVLM premotor neurons comprises adrenergic C1 cells that synthesize epinephrine, further contributing to blood pressure regulation. The RVLM’s activity is modulated by inhibitory inputs from the NTS, where baroreceptor-mediated signals are relayed via GABAergic neurons to suppress sympathetic excitation. Thus, the RVLM acts as a key integrative hub for maintaining cardiovascular homeostasis.

#### 3.2.3. Nucleus of the Solitary Tract (NTS)

NTS, located dorsally along the vagus nerve, serves as the principal sensory nucleus for visceral afferent inputs. It acts as the primary central terminal site for baroreceptor and chemoreceptor afferents carried by the glossopharyngeal (CN IX) and vagus (CN X) nerves from the carotid sinus and aortic arch [53]. After processing this peripheral visceral information, the NTS relays signals via second-order neurons that project to other key brain regions involved in autonomic control. A major efferent pathway from the NTS projects to the PVN, enabling hypothalamic integration of autonomic feedback. Additionally, the NTS sends direct projections to the RVLM. The RVLM, in turn, sends efferent fibers to the IML of the spinal cord. These IML neurons give rise to preganglionic sympathetic fibers that ultimately innervate the heart through a multi-synaptic pathway, critically influencing sympathetic nerve activity and blood pressure regulation.

**Figure 2:**
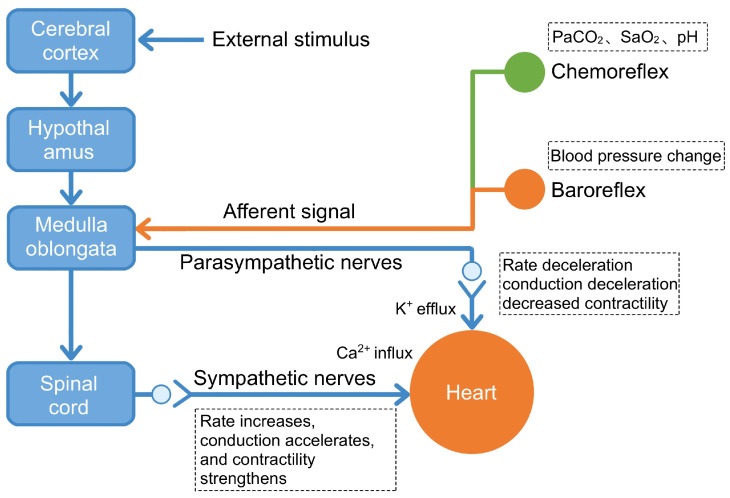
CNS network that regulates cardiac activity. This diagram illustrates the functional circuitry through which the central nervous system modulates cardiac performance via both descending efferent pathways and ascending afferent feedback loops. Descending control: The cerebral cortex processes external stimuli (e.g., stress, emotions) and relays signals through the hypothalamus to the medulla oblongata, which activates either sympathetic pathways (leading to increased heart rate, enhanced conduction, and strengthened contractility via Ca^2+^ influx) or parasympathetic pathways (vagus nerve, resulting in decreased heart rate, slowed conduction, and reduced contractility via K^+^ efflux). Ascending feedback: Baroreceptors detect blood pressure changes, while chemoreceptors monitor PaCO_2_, SaO_2_, and pH levels. These peripheral sensors transmit afferent signals to the medulla oblongata via the nucleus of the solitary tract, enabling dynamic correction of central autonomic commands. This bidirectional neurocardiac circuitry operates continuously under normal conditions to maintain cardiovascular homeostasis. Post-MI, disruption of these reflex loops—particularly reduced baroreceptor sensitivity and impaired vagal feedback—contributes to autonomic imbalance and arrhythmogenesis. Figure adapted from [54].

In summary, the NTS functions as a critical first-order integration center for cardiovascular and autonomic reflexes. By relaying visceral sensory information to both the PVN and the RVLM, it plays an indispensable role in the central circuitry governing sympathetic outflow. Consequently, the NTS is a pivotal region for understanding mechanisms underlying sympathetic remodeling in conditions such as MI and remains a key focus of current cardiovascular research (Figure 2).

## 4. Central Regulatory Mechanisms of Sympathetic Remodeling Following MI

The causal relationship between central mechanisms and peripheral arrhythmogenesis is established through multiple experimental approaches: (1) pathway-specific pharmacological inhibition or genetic deletion that reduces both neuroinflammation and arrhythmia burden; (2) temporal correlation between pathway activation (3–7 days post-MI) and peak arrhythmia vulnerability; (3) direct demonstration that interventions targeting central nuclei alter peripheral sympathetic innervation patterns and electrophysiological substrates; and (4) programmable electrical stimulation studies showing reduced arrhythmia inducibility following pathway modulation. These criteria distinguish causal mechanisms from mere associations.

Building upon the multi-level anatomical foundation, the pivotal mechanism driving post-MI sympathetic remodeling and subsequent malignant arrhythmias is a vicious cycle formed between neuroinflammation within the CNS and peripheral sympathetic overactivation.

While this review emphasizes sympathetic mechanisms, it is important to note that parasympathetic withdrawal and impaired vagal tone also contribute to post-MI arrhythmogenesis. The NTS receives vagal afferents and modulates both sympathetic (via projections to RVLM) and parasympathetic outflow. Post-MI, reduced vagal activity—reflected in decreased heart rate variability—removes protective anti-arrhythmic influences. The sympathetic-parasympathetic imbalance, rather than sympathetic excess alone, creates the final arrhythmogenic milieu. Therapeutic strategies that enhance vagal tone (e.g., vagal nerve stimulation) may therefore complement sympathetic neuromodulation.

VA are the most prevalent form of lethal rhythm disturbance post-MI and the primary cause of SCD. One underlying mechanism is reentry, typically initiated by persistent ventricular tachycardia. Following MI, myocardial necrosis and degeneration in the infarct zone leave behind islands of surviving myocytes embedded within scar tissue. The electrical conduction within these patches is significantly slowed compared to normal myocardium, creating a slow conduction zone at the scar border that serves as an anatomical substrate for reentrant circuits.

Conversely, post-MI cardiac sympathetic nerve sprouting and hyperinnervation lead to increased sympathetic tone. The excessive release of NE from these regenerated nerves is a critical mechanism underpinning the genesis and persistence of VA. Therefore, suppressing this pathological sympathetic activation is of paramount importance for reducing arrhythmia incidence. An acute inflammatory response after MI triggers neuroinflammation and oxidative stress within key central regulatory regions, such as the hypothalamic PVN. This is manifested as increased neuronal excitability, neural remodeling, and infiltration of inflammatory cells, which ultimately lead to the excitation of cardiac sympathetic nerves [55]. Microglia, the primary immune cells in the CNS, play a critical role in this process by mediating neuroimmune responses within the PVN.

The sympathetic dysregulation within the PVN involves the convergence of multiple signaling pathways. The TLR4/MyD88/NF-κB pathway mediates the initiation of neuroinflammation, while the P2X7R/NLRP3 inflammasome pathway acts to amplify the inflammatory cascade. Concurrently, weakened inhibitory control from neurotransmitters like GABA leads to disinhibition. Together, these elements form a self-perpetuating vicious cycle of “inflammatory activation-inhibition imbalance,” which constitutes the core mechanism of excessive sympathetic outflow after MI.

### 4.1. The Role of the TLR4/MyD88/NF-κB Signaling Pathway in the PVN in Post-MI Sympathetic Nerve Remodeling

Activation of microglia within the PVN and the associated neuroinflammatory response have been implicated in the development of VAs following MI. Toll-like receptor 4 (TLR4), which is notably upregulated in PVN microglia after MI, participates in this sympathetic overexcitation. Inhibition of TLR4 within the PVN has been shown to attenuate sympathetic hyperactivity, a effect partially attributed to the modulation of the NF-κB pathway and a reduction in oxidative stress [56].

TLR4, expressed broadly on CNS cells, is a key regulator of inflammatory cytokine release and microglial immune responses. NF-κB, in turn, acts as a critical transcription factor that promotes the activation of inflammatory cells and the production of reactive oxygen species (ROS). Following MI, the interplay between the TLR4/NF-κB axis and ROS generation is believed to enhance neuronal excitation and NE release within the CNS, ultimately increasing sympathetic outflow and contributing to adverse cardiac outcomes. Wang et al. demonstrated that NF-κB is highly activated at MI sites, triggering ROS production and fostering a pro-inflammatory state that promotes sympathetic nerve sprouting and hyperactivation [57]. The finding that TLR4 inhibition suppresses this sympathoexcitation underscores its potential as a therapeutic target for modulating NF-κB and oxidative stress in post-MI sympathetic remodeling [58].

**Figure 3:**
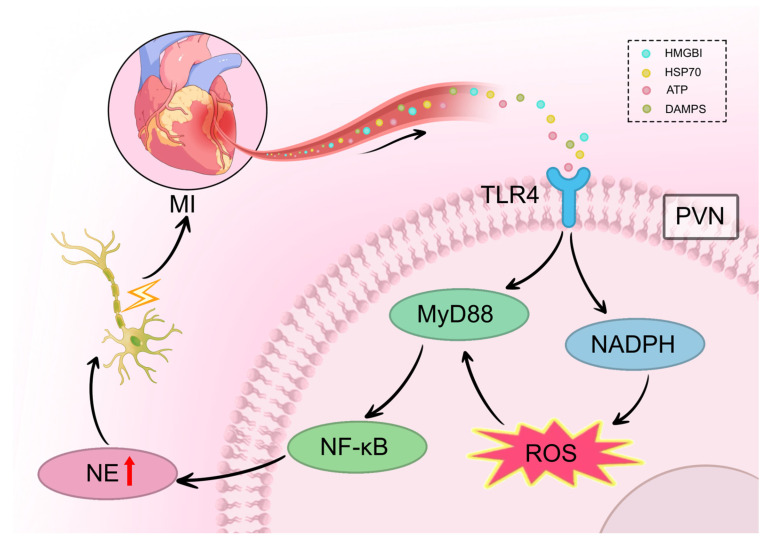
TLR4/MyD88/NF-κB pathway mediates neuroinflammation-induced sympathetic remodeling after MI. Following MI, danger-associated molecular patterns (HMGB1, HSP70, ATP, DAMPs) are released from infarcted myocardium and travel via the bloodstream to the brain. In the PVN, these signals activate TLR4 receptors on microglia, triggering MyD88-dependent recruitment and nuclear translocation of NF-κB. This signaling cascade activates NADPH oxidase, leading to ROS generation and the release of pro-inflammatory cytokines including IL-1β and TNF-α. The resulting neuroinflammation increases neuronal excitability within the PVN, facilitating sympathetic overactivation via multi-synaptic pathways (PVN → RVLM → IML → stellate ganglia). This ultimately drives excessive NE release from cardiac sympathetic nerve terminals, promoting heterogeneous sympathetic nerve sprouting in the heart. Symbol explanations: Lightning bolt (⚡): Indicates electrical neural signal transmission and increased neuronal excitability within the PVN and along the sympathetic pathway. Red upward arrow (↑) within NE vesicle: Indicates increased norepinephrine synthesis and release. Abbreviations: ATP, adenosine triphosphate; DAMPs, damage-associated molecular patterns; HMGB1, high mobility group box 1; HSP70, heat shock protein 70; MI, myocardial infarction; MyD88, myeloid differentiation primary response 88; NADPH, nicotinamide adenine dinucleotide phosphate; NE, norepinephrine; NF-κB, nucear factor kappa B; PVN, paraventricular nucleus; ROS, reactive oxygen species; TLR4, Toll-like receptor 4. Figure is original artwork created for this review.

The TLR4/MyD88/NF-κB signaling pathway is a well-established mediator of neuroinflammation in various CNS disorders. In the context of cardiovascular disease, this pathway also contributes to cardiomyocyte injury, fibrosis, and heart failure post-MI. Yang et al. reported that the TLR4/MyD88/NF-κB pathway is upregulated in the RVLM in a model of stress-induced hypertension, where it promotes neuroinflammation, increases sympathetic activity, and elevates blood pressure [59]. Similarly, after MI, elevated levels of TLR4 and MyD88, along with increased nuclear translocation of NF-κB, are observed in the PVN. Inhibition of TLR4 suppresses the expression of these mediators, attenuates sympathetic activation and remodeling, and improves cardiac function [59]. It is hypothesized that MI-induced activation of the TLR4/MyD88/NF-κB pathway in PVN microglia initiates a neuroimmune response, driving central and peripheral sympathetic excitation that culminates in malignant VAs (Figure 3).

Furthermore, emerging evidence highlights the role of N6-methyladenosine (m6A) methylation, a prevalent mRNA modification, in regulating inflammatory processes. Experimental data indicate that METTL3, the primary m6A methyltransferase predominantly located in microglia, is significantly enhanced in the PVN three days after MI. This increase promotes m6A modification on the 3′-UTR of TLR4 mRNA, synergizing with NF-κB signaling to amplify the production of pro-inflammatory cytokines such as IL-1β and TNF-α. This METTL3-mediated m6A-dependent mechanism further exacerbates sympathetic hyperactivity and elevates the risk of VAs post-MI [60].

The causal relationship between TLR4/MyD88/NF-κB activation and ventricular arrhythmias has been established through several lines of evidence. First, pharmacological TLR4 inhibition in the PVN not only reduces sympathetic nerve density in the peri-infarct zone but also significantly decreases the inducibility of ventricular tachycardia during programmed electrical stimulation in animal models [46,49]. Second, the temporal profile of TLR4 activation in the PVN (peaking at 3–7 days post-MI) correlates with the window of highest arrhythmia risk. Third, the excessive NE release driven by this pathway directly prolongs ventricular action potential duration and increases spatial dispersion of repolarization—the immediate electrophysiological substrate for reentrant arrhythmias [59]. These mechanistic links demonstrate that TLR4-mediated neuroinflammation is not merely associated with arrhythmias but represents a direct causal pathway from central immune activation to peripheral arrhythmogenic remodeling.

### 4.2. The Role of P2X7 Receptor Activation in the PVN in Post-MI Sympathetic Neural Remodeling

Myocardial injury following acute MI is exacerbated by a local neuroinflammatory response triggered by pro-inflammatory cytokines such as tumor necrosis factor-α (TNF-α), interferon-γ, and interleukin-1β (IL-1β). Among these, IL-1β is regarded as a pivotal mediator of neuroinflammation. It is primarily released by activated monocytes and macrophages and plays a central role in regulating the synthesis of nerve growth factor (NGF) within non-neuronal cells of peripheral nerves. The NOD-like receptor family, pyrin domain containing 3 (NLRP3) inflammasome, a multiprotein complex widely studied in recent years, promotes inflammatory and apoptotic processes by regulating caspase-1 activation, which in turn induces the maturation and secretion of IL-1β and other inflammatory factors. The P2X7 receptor (P2X7R) has been shown to critically influence IL-1β secretion via activation of the downstream NLRP3 inflammasome [61].

As a member of the ATP-gated non-selective cation channel P2X receptor family, P2X7R is widely expressed throughout the body, with prominent presence on hematopoietic-derived cells such as macrophages and microglia. This receptor is primarily activated by elevated extracellular ATP levels, which occur under conditions of cellular injury, hypoxia, or mechanical stress. Receptor activation triggers the release of ROS and promotes nuclear factor kappa-B (NF-κB) signaling at sites of tissue damage [62]. Experimental studies in mouse AMI models have detected increased inflammasome expression and enhanced caspase-1 activity in cardiomyocytes within the infarct and border zones; the administration of P2X7R inhibitors was found to reduce apoptosis and ameliorate adverse ventricular remodeling, underscoring the role of P2X7R in activating the NLRP3 pathway post-AMI [63].

P2X7R has emerged as a significant therapeutic target in cardiovascular disease due to its key involvement in post-infarction inflammation and neural remodeling. It promotes sympathetic hyperinnervation and arrhythmogenesis largely through the NLRP3/IL-1β axis. Inhibition of P2X7R has been demonstrated to attenuate sympathetic nerve sprouting and lower the incidence of VA [64]. Macrophages, by secreting NGF, form a critical link between the immune and nervous systems, presenting a potential target for modulating cardiac sympathetic overinnervation. Although the precise mechanisms remain under investigation, evidence suggests that P2X7R influences NGF-related sympathetic hyperinnervation via IL-1β.

Within the PVN of the hypothalamus, microglial activation contributes significantly to cytokine release via upregulated P2X7R expression. P2X7R activation induces Ca^2+^ influx and K^+^ efflux, facilitating caspase-1-dependent IL-1β maturation and release. Consistent with this, microinjection of TNF-α and IL-1β into the rat PVN has been shown to enhance peripheral sympathetic excitability [8].

Studies confirm that P2X7R is upregulated in microglia-derived neuroinflammation and that its overexpression stimulates microglial activation and proliferation. In a rat MI model, Cheng et al. reported a significant increase in the number of microglia and elevated levels of IL-1β mRNA and protein in the PVN of AMI rats compared to controls [65]. Further investigation revealed that AMI-induced activation of NADPH oxidase 2 (NOX2) in PVN microglia led to ROS overproduction, increased NE levels, and sympathetic hyperactivity. Treatment with the P2X7R antagonist Brilliant Blue G suppressed microglial activation, reduced NOX2 and ROS expression, and attenuated neuroinflammation, ultimately mitigating sympathetic overactivation and improving cardiac function [66]. The involvement of multiple central nuclei in post-MI sympathetic regulation is well-recognized. The NTS, for instance, acts as a primary relay for cardiac afferent signals, which are then transmitted to higher centers like the PVN. Activation of microglial P2X7R in the PVN leads to the release of pro-inflammatory cytokines, which in turn stimulate oxytocinergic and vasopressinergic neurons, thereby augmenting sympathetic nerve activity [67].

**Figure 4:**
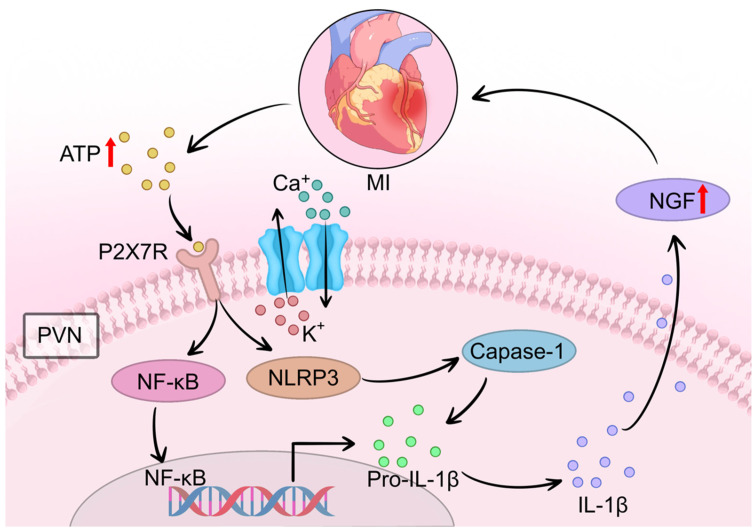
The P2X7R/NLRP3 pathway amplifies neuroinflammation and drives NGF-mediated sympathetic sprouting after MI. Following MI, extracellular ATP released from damaged cardiomyocytes activates microglial P2X7R in the PVN. This activation triggers calcium (Ca^2+^) influx and potassium (K^+^) efflux, leading to NOX2-mediated ROS generation and subsequent NLRP3 inflammasome assembly. The inflammasome promotes caspase-1-dependent cleavage of pro-IL-1β to mature IL-1β. Released IL-1β activates NF-κB signaling, which in turn stimulates NGF synthesis in cardiac macrophages. Elevated NGF levels directly drive excessive sympathetic nerve sprouting in the infarcted and peri-infarct myocardium. Concurrently, P2X7R activation excites vasopressin (VN) and oxytocin neurons in the PVN, further enhancing central sympathetic outflow to the heart. This pathway represents a critical “amplification phase” that sustains sympathetic remodeling and contributes to arrhythmogenesis. Symbol explanations: Black arrows: Indicate the direction of signaling cascades, ion fluxes, and molecular interactions.Red upward arrows (↑): Represent increased levels, upregulation, or enhanced release of molecules (ATP release from damaged cardiomyocytes, NGF upregulation, and norepinephrine synthesis/release). Abbreviations: ATP, adenosine triphosphate; Ca^2+^, calcium ions; Caspase-1, cysteine-aspartic acid protease 1; IL-1β, interleukin-1 beta; K^+^, potassium ions; MI, myocardial infarction; NF-κB, nuclear factor kappa B; NGF, nerve growth factor; NLRP3, NOD-like receptor family pyrin domain containing 3; P2X7R, P2X7 receptor; Pro-IL-1β, pro-interleukin-1 beta; PVN, paraventricular nucleus. Figure is original artwork created for this review.

In summary, a body of evidence confirms that ATP-induced P2X7R upregulation in microglia is closely associated with neuroinflammatory responses and neuronal excitation post-AMI. Elevated levels of ATP and IL-1β in the PVN drive P2X7R-mediated microglial activation and ROS overproduction. Activated microglia release a plethora of inflammatory mediators—including cytokines, chemokines, ROS, TNF-α, and IL-6—which propagate excitatory signals to PVN regions governing sympathetic outflow. This cascade results in sustained sympathetic overactivity and exacerbates cardiac dysfunction, forming a vicious cycle of neurocardiogenic injury (Figure 4).

The P2X7R/NLRP3 pathway’s contribution to ventricular arrhythmias extends beyond neuroinflammation to direct effects on cardiac electrophysiology. Experimental evidence demonstrates that P2X7R antagonism reduces not only sympathetic nerve sprouting density but also decreases premature ventricular contraction burden and suppresses inducible ventricular tachycardia. The mechanism involves NGF-mediated hyperinnervation specifically targeting the infarct border zone, creating spatially heterogeneous sympathetic innervation that facilitates triggered activity. Furthermore, IL-1β released through NLRP3 inflammasome activation sensitizes cardiac neurons to catecholamines, lowering the threshold for arrhythmia initiation [65,66]. This pathway thus represents an “amplification mechanism” that transforms initial ischemic injury signals into sustained arrhythmogenic sympathetic remodeling. Importantly, the P2X7R pathway appears necessary for maintaining chronic sympathetic hyperactivity, as late-phase P2X7R inhibition (weeks post-MI) can still reverse established sympathetic remodeling and reduce arrhythmia incidence.

### 4.3. GABAergic Inhibition in the PVN Attenuates Sympathetic Hyperinnervation Post-MI

GABA serves as the primary inhibitory neurotransmitter in the CNS. Within the PVN, GABAergic afferent inputs originate predominantly from local hypothalamic sources and regions adjacent to the dorsal and lateral borders of the PVN. Both ionotropic GABA-A receptors and metabotropic GABA-B receptors are abundantly expressed on PVN neurons. GABA mediates two complementary forms of inhibition over the activity of presympathetic neurons projecting to the PVN-RVLM pathway: (1) a phasic inhibition via classical synaptic transmission, generating inhibitory postsynaptic currents; and (2) a tonic inhibition mediated by persistent activation of extrasynaptic GABA-A receptors, resulting in a sustained tonic inhibitory current (Itonic) that modulates neuronal excitability [68].

Research on tonic inhibition in PVN-RVLM sympathetic neurons has highlighted the role of the GABA transporter 3 (GAT-3) located on astrocytes. However, the precise regulatory mechanism remained incompletely understood [69]. Subsequent investigations in cardiovascular disease models revealed that the tonic inhibition of PVN-RVLM neurons is significantly attenuated in rats with heart failure (HF) following MI, compared to sham-operated (SHAM) controls. This impairment was associated with enhanced GABA uptake by astrocytic GAT-3 transporters within the PVN. Critically, application of a selective GAT-3 blocker restored tonic inhibition, indicating that increased GAT-3 expression after MI reduces extracellular GABA levels in the PVN-RVLM pathway. This diminishment of GABAergic tone leads to disinhibition of PVN neurons, thereby contributing to sympathetic hyperactivity observed post-MI [70,71].

**Figure 5:**
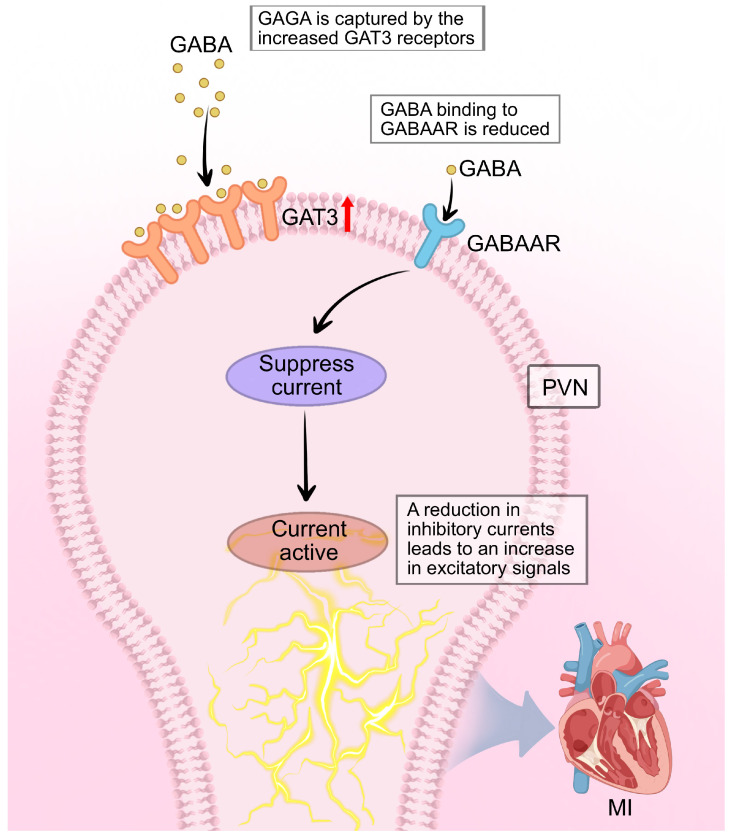
GABAergic disinhibition in the PVN contributes to sympathetic hyperactivity after MI. Under physiological conditions, tonic inhibitory current (Itonic) generated by GABA acting on extrasynaptic GABAAR maintains presympathetic PVN neurons projecting to the RVLM within a normal activity range. Following MI, astrocytic GAT-3 is upregulated, enhancing GABA reuptake from the synaptic cleft and reducing extracellular GABA concentration. This leads to a decrease in Itonic, resulting in disinhibition of PVN-RVLM projection neurons. The loss of GABAergic restraint unmasks and potentiates excitatory glutamatergic inputs, elevating sympathetic outflow to the heart. Symbol explanations: Black arrows: Indicate the direction of neural signaling and GABA transport. Red upward arrow (↑) on GAT-3: Represents upregulation of GAT-3 expression post-MI. Orange receptor symbols: Represent GAT-3 transporter proteins. Blue receptor symbols: Represent GABAAR. Abbreviations: GABA, gamma-aminobutyric acid; GABAAR, GABA-A receptor; GAT-3, GABA transporter 3; MI, myocardial infarction. Figure is original artwork created for this review.

Supporting this, Mendonça et al. demonstrated that microinjection of the GABA-A receptor agonist muscimol into the PVN inhibits neuronal activity under normal conditions, suppresses sympathetic nerve activity, promotes parasympathetic activity, and mitigates adverse cardiovascular outcomes induced by sympathetic overexcitation [72]. Conversely, blockade of GABA-A receptors within the PVN elicits pressor responses, positive chronotropy, and positive inotropy, reflecting sympathetic activation. Collectively, these findings indicate that in the context of post-MI HF, the inhibitory influence of GABA on sympathetic outflow is substantially reduced. This impaired GABAergic inhibition results in neuronal disinhibition within the PVN, which is a key mechanism underpinning the increased sympathetic drive that characterizes heart failure (HF) [73] (Figure 5).

The loss of GABAergic inhibition directly enables arrhythmogenesis through a “disinhibition mechanism.” Under normal conditions, tonic GABA inhibition acts as a critical brake on PVN-RVLM neuronal activity, maintaining sympathetic outflow within physiological limits. Post-MI, the upregulation of astrocytic GAT-3 reduces extracellular GABA, effectively removing this brake and allowing excitatory glutamatergic inputs to dominate. This disinhibition not only increases baseline sympathetic tone but, critically, amplifies sympathetic responses to stress and other triggers—explaining why arrhythmias often occur during emotional or physical stress in post-MI patients. Experimental restoration of GABAergic tone through GAT-3 blockade or GABA-A receptor agonists reduces both spontaneous ventricular arrhythmias and stress-induced arrhythmia vulnerability [72,73]. This demonstrates that GABAergic disinhibition is not merely correlated with but is causally required for the full expression of sympathetic-driven arrhythmogenesis post-MI.

### 4.4. Peripheral Amplification: Stellate Ganglion Remodeling

Central sympathetic dysregulation is amplified at the stellate ganglion (SG) level through local cellular and molecular changes. Post-MI, SG postganglionic neurons undergo hypertrophy and exhibit increased excitability [20]. Satellite glial cells, normally providing metabolic support, become activated and participate in neuroimmune signaling through the P2Y1R/IGFBP2 pathway [39]. ATP released from damaged cardiac tissue activates glial P2Y1 receptors, stimulating IGFBP2 release that promotes neuronal sprouting. This creates a peripheral amplification loop operating semi-independently of central control.

Extracellular matrix remodeling within the SG parallels cardiac changes: loss of chondroitin sulfate proteoglycans removes inhibitory signals for axonal growth, enabling aberrant sympathetic fiber sprouting [34]. Temporally, SG activation peaks during the acute inflammatory phase (3–7 days post-MI) and sustains through chronic remodeling, mirroring central neuroinflammatory dynamics.

The integration of central and peripheral mechanisms forms a complete circuit: PVN neuroinflammation (TLR4, P2X7R, GABA pathways) → RVLM amplification → spinal IML relay → stellate ganglion (further amplified by P2Y1R/IGFBP2 and ECM changes) → heterogeneous cardiac innervation → arrhythmogenic substrate. Clinical interventions targeting the SG—including stellate ganglion block (60–70% acute efficacy) and surgical denervation—validate its critical role in this circuit [18,19]. 

Table 1 summarizes the three major central pathways mediating post-MI sympathetic remodeling, including their experimental models, key mechanistic findings, and effects on arrhythmogenesis.

## 5. Clinical Translation and Future Perspectives

### 5.1. Neuromodulation Strategies

Stellate ganglion block (SGB) using local anesthetics provides temporary sympathetic suppression, achieving 60–70% acute arrhythmia control in electrical storm. Cardiac sympathetic denervation (CSD) via surgical or minimally invasive approaches offers sustained arrhythmia reduction in patients with refractory ventricular tachycardia [18]. Vagal nerve stimulation (VNS) enhances parasympathetic tone and shows promise in heart failure patients. Non-invasive approaches including transcutaneous vagal stimulation warrant further investigation. Optimization of patient selection, timing, and technique remains a priority.

### 5.2. Molecular Therapeutic Targets

Preclinical studies demonstrate that TLR4 antagonists, P2X7R inhibitors, and NLRP3 inflammasome blockers reduce both PVN neuroinflammation and peripheral sympathetic remodeling. GAT-3 inhibition offers potential to restore GABAergic tone. Clinical translation requires development of brain-penetrant compounds with acceptable safety profiles. Repurposing existing medications with relevant properties (e.g., statins for TLR4 inhibition) may accelerate pathway-to-clinic timelines.

### 5.3. TBiomarkers for Risk Stratification

Heart rate variability (HRV) analysis, particularly advanced metrics like deceleration capacity, provides non-invasive autonomic assessment with prognostic value (clinically validated). Cardiac sympathetic imaging using ^123^I-MIBG scintigraphy visualizes innervation patterns and predicts arrhythmia risk independent of ejection fraction (clinical evidence). Circulating biomarkers including neuropeptide Y (NPY), IL-1β, and TNF-α show promise for identifying active neuroimmune activation (largely preclinical). Integration of these modalities could enable personalized risk stratification.

### 5.4. Distinguishing Clinical from Preclinical Evidence

Most mechanistic insights (TLR4, P2X7R, GABA pathways) derive from rodent MI models. While these identify key molecular targets, human validation through CSF biomarker studies, neuroimaging, and clinical trials with mechanistic endpoints is needed. Stellate ganglion interventions have clinical evidence, whereas molecular pathway inhibitors remain preclinical. Bridging this gap requires trials incorporating both clinical outcomes (arrhythmia burden) and mechanistic measures (sympathetic imaging, autonomic biomarkers).

### 5.5. Research Priorities and Clinical Implementation

Key priorities include (1) validating central neuroimmune mechanisms in human post-MI cohorts; (2) phase II trials of pathway-specific inhibitors with arrhythmia endpoints; (3) comparative effectiveness studies of neuromodulation approaches; (4) large animal models to bridge translational gaps; and (5) precision medicine strategies using genomic profiling. Near-term implementation should incorporate HRV and MIBG imaging into routine risk assessment, expand SGB availability for electrical storm, and develop multidisciplinary neurocardiology programs.

For forensic medicine, systematic evaluation of sympathetic innervation patterns, central autonomic nuclei, and stellate ganglion histopathology in sudden cardiac death cases could identify neuroimmune-mediated mechanisms, improving diagnostic accuracy and family counseling.

## 6. Conclusions

Sympathetic remodeling following MI involves coordinated changes across the brain–heart axis. Three PVN signaling pathways—TLR4/MyD88/NF-κB, P2X7R/NLRP3, and GABAergic disinhibition—initiate and amplify central sympathetic dysregulation. Peripheral stellate ganglion remodeling (P2Y1R/IGFBP2, ECM changes) further amplifies these signals, producing heterogeneous cardiac hyperinnervation that creates arrhythmogenic substrates. Causal links between molecular pathways and arrhythmias are established through interventional studies showing that pathway modulation reduces both neuroinflammation and arrhythmia burden. Emerging biomarkers and neuromodulation strategies offer pathways toward mechanism-based prevention, but rigorous clinical validation is essential to translate preclinical discoveries into improved patient outcomes.

## Figures and Tables

**Table 1:** Summary of central pathways in post-MI sympathetic remodeling.

Pathway	Model	Key Findings	Effect on Arrhythmias
TLR4/MyD88/NF-κB	Rat MI (LAD ligation)	TLR4 activation → NF-κB translocation → inflammatory cytokine release (IL-1β, TNF-α)	Increases VT inducibility; TLR4 inhibition reduces arrhythmias
P2X7R/NLRP3	Rat MI (LAD ligation)	ATP → P2X7R activation → NLRP3 inflammasome → IL-1β release → NGF upregulation	P2X7R blockade reduces PVCs and VT; decreases nerve sprouting
GABA Disinhibition	Rat HF post-MI	Astrocytic GAT-3 upregulation → reduced GABA → loss of tonic inhibition	Increases stress-triggered arrhythmias; GAT-3 blockade restores inhibition

Abbreviations: GABA, gamma-aminobutyric acid; GAT-3, GABA transporter 3; HF, heart failure; IL-1β, interleukin-1β; LAD, left anterior descending artery; MI, myocardial infarction; NGF, nerve growth factor; PVC, premature ventricular contraction; VT, ventricular tachycardia.

## Data Availability

No new data were created or analyzed in this study. Data sharing is not applicable to this article.

## Abbreviations

The following abbreviations are used in this manuscript:

ACh	Acetylcholine
APD	Action potential duration
ATP	Adenosine triphosphate
CNS	Central nervous system
CSD	Cardiac sympathetic denervation
CSPG	Chondroitin sulfate proteoglycan
ECM	Extracellular matrix
GABA	Gamma-aminobutyric acid
GAT-3	GABA transporter 3
GP	Ganglion plexi
HF	Heart failure
ICNS	Intrinsic cardiac nervous system
IGFBP2	Insulin-like growth factor binding protein 2
IL-1β	Interleukin-1 β
IML	Intermediolateral cell column
Itonic	Tonic inhibitory current
LAD	Left anterior descending coronary artery
m6A	N6-methyladenosine
MI	Myocardial infarction
MIBG	Metaiodobenzylguanidine
NF-κB	Nuclear factor κB
NGF	Nerve growth factor
NLRP3	NOD-like receptor family, pyrin domain-containing protein 3
NOX2	NADPH oxidase 2
NTS	Nucleus of the solitary tract
P2X7R	P2X7 receptor
PVC	Premature ventricular contraction
PVN	Paraventricular nucleus
ROS	Reactive oxygen species
RVLM	Rostral ventrolateral medulla
SCD	Sudden cardiac death
SGB	Stellate ganglion block
TLR4	Toll-like receptor 4
TNF-α	Tumor necrosis factor -α
VAs	Ventricular arrhythmias
VNS	Vagal nerve stimulation
VT	Ventricular tachycardia
